# Outcomes of endovascular embolization for Vein of Galen malformations: An individual participant data meta-analysis

**DOI:** 10.3389/fped.2022.976060

**Published:** 2022-09-30

**Authors:** Cody Savage, Andrew T. Hale, Matthew S. Parr, Alexander Hedaya, Benjamin W. Saccomano, Georges Bouobda Tsemo, Muhammad U. Hafeez, Omar Tanweer, Peter Kan, Laurent J. Solomon, Dan Meila, Peter B. Dirks, Jeffrey P. Blount, James M. Johnston, Brandon G. Rocque, Curtis J. Rozzelle, Kartik Bhatia, Prakash Muthusami, Timo Krings, Jesse Jones

**Affiliations:** ^1^ Heersink School of Medicine, University of Alabama at Birmingham, Birmingham, AL, United States; ^2^ Department of Neurosurgery, University of Alabama at Birmingham, Birmingham, AL, United States; ^3^ Department of Neurology, University of Alabama at Birmingham, Birmingham, AL, United States; ^4^ Department of Neurology, Baylor College of Medicine, Houston, TX, United States; ^5^ Department of Neurosurgery, Baylor College of Medicine, Houston TX, United States; ^6^ Department of Neurosurgery, University of Texas Medical Branch at Galveston, Galveston, TX, United States; ^7^ Department of Obstetrics and Fetal Medicine, Paris Descartes University, Assistance Publique-Hôpitaux de Paris, Hôpital Necker Enfants, Paris, France; ^8^ Department of Interventional Radiology, Helois Klinikum Krefeld, Johanna-Etienne Hospital Neuss, Neuss, Germany; ^9^ Division of Pediatric Neurosurgery, The Hospital for Sick Children, Toronto, ON, Canada; ^10^ Department of Medical Imaging, Sydney Children’s Hospital Network, Westmead, NSW, Australia; ^11^ Division of Interventional Radiology, University of Toronto and the Hospital for Sick Children, Toronto, ON, Canada

**Keywords:** Vein of Galen aneurysmal malformation, endovascular embolization, meta-analysis, congenital vascular anomaly, pediatric

## Abstract

**Introduction:**

Understanding outcomes after Vein of Galen malformation (VOGM) embolization has been limited by small sample size in reported series and predominantly single center studies. To address these limitations, we perform an individual-participant meta-analysis (IPMA) to identify risk factors associated with all-cause mortality and clinical outcome after VOGM endovascular embolization.

**Methods:**

We performed a systematic review and IPMA of VOGM endovascular outcomes according to PRISMA guidelines. Individual patient characteristics including demographic, intra/post-operative adverse events, treatment efficacy (partial or complete occlusion), and clinical outcome were collected. Mixed-effects logistic regression with random effects modeling and Bonferroni correction was used (*p* ≤ 0.003 threshold for statistical significance). The primary and secondary outcomes were all-cause mortality and poor clinical outcome (moderate/severe developmental delay or permanent disabling injury), respectively. Data are expressed as (mean ± standard deviation (SD)) or (odds ratio (OR), 95% confidence interval (CI), *I*^2^, *p*-value)

**Results:**

Thirty-five studies totaling 307 participants quantifying outcomes after endovascular embolization for VOGM were included. Follow up time was 42 (±57) months. Our analysis contained 42% neonates (<1 month) at first embolization, 45% infants (1 month ≤2 years), and 13% children (>2 years). Complete occlusion was reported in 48% of participants. Overall all-cause mortality was 16%. Overall, good clinical outcome was achieved in 68% of participants. First embolization as a neonate [OR = 6.93; 95% CI (1.99–24.08); *I*^2^ < 0.01; *p* < 0.001] and incomplete embolization [OR = 10.87; 95% CI (1.86–63.55); *I*^2^ < 0.01; *p* < 0.001] were associated with mortality. First embolization as a neonate [OR = 3.24; 95% CI (1.47–7.15); *I*^2^ < 0.01; *p* < 0.001], incomplete embolization [OR = 5.26; 95% CI (2.06–13.43); *I*^2^ < 0.01; *p* < 0.001], and heart failure at presentation [OR = 3.10; 95% CI (1.03–9.33); *I*^2^ < 0.01; *p* = 0.002] were associated with poor clinical outcomes. Sex, angioarchitecture of lesion, embolization approach (transvenous vs. transarterial), and single or multistage embolization were not associated with mortality or clinical outcome.

**Conclusions:**

We identify incomplete VOGM embolization independently associated with mortality and poor clinical outcome. While this study provides the highest level of evidence for VOGM embolization to date, prospective multicenter studies are needed to understand the optimal treatment strategies, outcomes, and natural history after VOGM embolization.

## Introduction

Vein of Galen aneurysmal malformations (VOGMs) are among the most common cerebrovascular arteriovenous malformations (AVM) in neonates and infants, accounting for ∼30% of neurovascular anomalies in this age group (1). VOGMs are the result of a shunt between the choroidal arteries and the median prosencephalic vein of Markowski (2). The overall incidence is low, with previous studies reporting <1/25,000 deliveries (3, 4). If left untreated, VOGMs have a nearly 100% mortality rate in childhood (5). While previous treatment strategies involved open neurosurgical repair, endovascular embolization is now the preferred treatment option due to improved mortality rates and clinical outcomes (6–12). Much of our foundational understanding and development of endovascular embolization for VOGM was established by the work of Lasjaunias and colleagues (3, 9, 13–34). In the largest study to date of endovascular embolization of VOGMs in 216 patients, Lasjaunias et al demonstrated a 10.6% mortality rate following embolization and a 17% adverse event rate, with 4% resulting in permanent neurological disability or death (9). They also reported 15.6% and 10.4% of the surviving 193 patients were moderately and severely intellectually disabled. Similar results were found in a recent meta-analysis where Yan et al. found an all-cause mortality rate and adverse event rate of 10% and 37%, respectively (35). The remaining 74% were developmentally appropriate at 12 months follow up. This finding was recapitulated in a separate meta-analysis, concluding that 62% of patients achieved a good neurological outcome (36). However, these analyses relied on aggregate, summary data and were not performed using individual patient data (IPD).

While embolization is the current treatment paradigm for VOGM, there is limited quantitative data on factors associated with clinical outcomes. Two meta-analyses have been performed to address this gap in knowledge (35, 36). Of the two, only Brinjikji et al. (36) examined predictors of clinical outcomes following endovascular embolization. In their analysis they stratified their participants into groups by age (unclear if at age of diagnosis or treatment): neonates (<1 month), infants (≥1 month to <2 years), and children (≥2 years), similar to a prior meta-analysis by Yan et al. (35). Utilizing aggregate data from 27 studies (578 participants), they found higher rates of poor neurologic outcome among neonates compared to infants. They also found participants presenting with congestive heart failure had lower rates of good neurologic outcomes. While these studies have been very informative, they were not able to delineate patient-level factors due to limitations of their study design. Thus, our aim was to perform the first individual-participant meta-analysis (IPMA), the gold-standard for meta-analysis (37), to identify risk factors associated with clinical outcomes after VOGM endovascular embolization.

## Methods

### Inclusion and exclusion criteria

Eligibility was determined by the following criteria: (1) any study (e.g., cohort, case-control, case series) where participants with VOGM underwent endovascular embolization, (2) the study was focused on outcomes and/or adverse events of endovascular embolization in VOGM, (3) text was available in English. Exclusion criteria included (1) studies where patient-level VOGM outcomes could not be determined, (2) case series with fewer than 2 treated participants, (3) review articles, (4) participants were the same population as another study, and where there was (5) spontaneous thrombosis of the lesion.

### Search strategy

Our study followed the Preferred Reporting Items for Systematic Reviews and Meta-Analyses (PRISMA) guidelines (38). The retrieval date of the electronic databases was searched until October 2021, with no restriction on the year of publication. Two authors (C.S, A.H) performed title and abstract screening, full text review, and data extraction independently. The two reviewers independently evaluated the selected full-text articles for inclusion in the systematic review. The reasons for exclusion during the screening of full-text articles were recorded. Repeat sampling was monitored for during the abstract screening and the full-text review. If repeat sampling occurred, only the article with the largest number of participants was included. Any disagreements on study eligibility were resolved by discussion with A.T.H. The primary and secondary outcomes were all-cause mortality and poor clinical outcome (moderate/severe developmental delay or permanent disabling injury), respectively.

Two authors (C.S, A.H) searched five electronic databases (PubMed, MEDLINE, EMBASE, CINAHL, and Web of Science) for relevant articles using the following terms: *vein of galen malformations or galen vein aneurysm or vein of galen aneurysm malformation or ectasia or varix of the vein of galen or galenic arteriovenous malformation or vein of galen malformation or malformations veins, galen or malformations vein, galen or vein of galen malformation and endovascular procedures or angioplasty or angioplasty, balloon or angioplasty, laser or atherectomy or angioscopy or catheterization, central venous or catheterization, peripheral or embolization, therapeutic or embolotherapy or embolotherapies or therapeutic embolizations or therapeutic embolization or embolizations, therapeutic*.

### Data extraction

Data from each study was independently extracted by two reviewers (C.S, A.H). If IPD could not be determined, the corresponding author was contacted and invited to participate in our study. Institutional Review Board (IRB) approval was obtained. The following was extracted from each study: (1) participant demographics, (2) presenting symptoms, (3) angioarchitecture of the VOGM (mural, choroidal, or mixed), (4) embolization vascular access route (transvenous, transarterial, umbilical, a combination thereof), (5) age(s) at embolization(s), (6) intra/post-operative adverse events after embolization(s), (7) treatment efficacy (partial or complete occlusion), (8) clinical outcome (poor clinical outcome defined as moderate/severe developmental delay or permanent disabling injury), (10) mortality, (11) and follow-up time (if available).

### Statistical analyses

Statistics were performed by M.S.P. Meta analysis was performed IPD in a single-stage approach. A random-effects logistic regression model, paneled by study, was utilized to test the association with each factor and the primary and secondary outcome measures: all-cause mortality and poor clinical outcome, respectively, as defined above. A Bonferroni correction for multiple comparisons was utilized, with a study-wide threshold of *p* ≤ 0.003 for statistical significance. Odds ratios with Bonferroni corrected 95% confidence intervals were calculated. Forest plots were constructed. Heterogeneity for each tested association was assessed with *I*^2^ statistic. *I*^2^ values <25%, 20%–50%, and >50% indicate heterogeneity is low, moderate, and high, respectively (39). Statistical analysis was performed using Stata/SE 17.0 (StataCorp, 2021, College Station, TX).

## Results

### Study selection and characteristics

**Figure 1:**
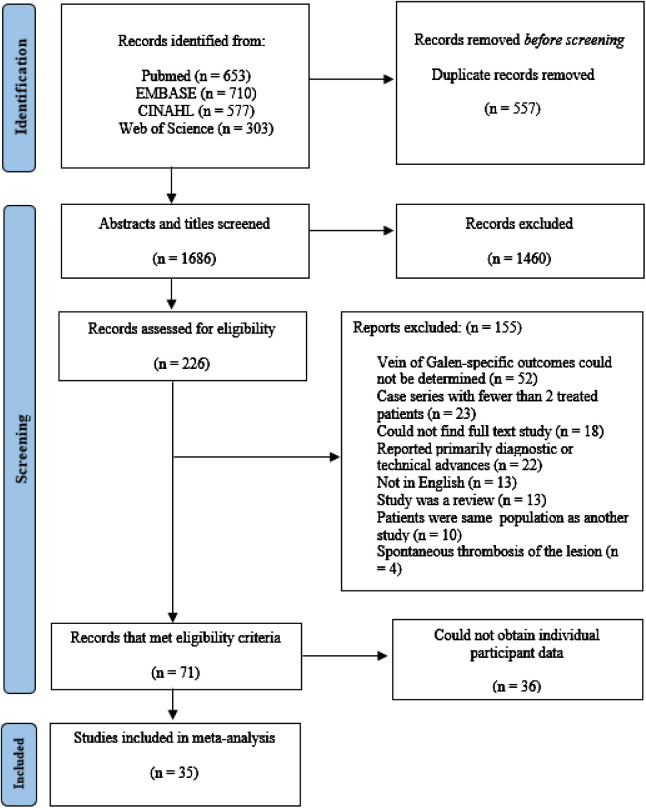
PRISMA flow diagram.

The results of our literature search are summarized in Figure 1. A total of 2,243 abstracts and titles were identified using our electronic search. Of these, 532 were removed as duplicates. Of the 1,713 studies that were screened, 266 were selected for full-text review. Of the 266, 71 met all inclusion criteria. A complete list of excluded studies with reasons for exclusion is available from the authors upon request. IPD was obtained in 35 of the eligible 71 articles (49%) (4, 6, 8, 17, 23, 28, 33, 40–66). This includes 2 (6%) articles containing 40 total participants that we obtained IPD after contacting the corresponding authors (10, 64). The study characteristics and total number of patients with IPD is summarized in Supplementary Table S1.

### Participant characteristics and follow-up

Information of the sex and age at first embolization was obtained in 57% (176/307) and 96% (296/307) of participants, respectively. Participants were divided into 3 groups by their age at first embolization: neonates (<1 month), infants (≥1 month to <2 years), and children (≥2 years), consistent with prior studies of VOGM (35, 36). Within this group, 42% were neonates (124/296), 45% were infants (132/296), and 13% were children (40/296) at first embolization (Table 1). Data on presenting symptoms was available for 68% (210/307) of participants. The three most common presenting symptoms were heart failure (68%; 142/210), hydrocephalus (15%; 31/210), and increasing head circumference (6%; 13/210). Follow-up data was obtained in 60% (184/307) of participants. In this group, the mean follow up was 42 (±57, SD) months. Other patient characteristics such as sex, angioarchitecture of lesion (e.g., choroidal, mural, or mixed), and vascular access route utilized during embolization is summarized in Table 1.

**Table 1:** Patient characteristics and adverse events.

Variable	*N* (%)	Total
Sex		176
Female	58 (33)	
Male	118 (67)	
Age group		296
Neonate	123 (42)	
Infant	132 (45)	
Child	40 (13)	
Presenting symptoms		210
Heart failure	142 (68)	
Hydrocephalus	31 (15)	
Increasing head circumference	13 (6)	
Seizure	6 (3)	
Prenatal ultrasonic diagnosis	4 (2)	
Intracranial hemorrhage	4 (2)	
Headache	3 (1)	
Other neurologic symptoms	3 (1)	
Facial pain	2 (1)	
Incidental finding	2 (1)	
Angioarchitecture		268
Choroidal	164 (61)	
Mural	89 (33)	
Mixed	15 (6)	
Access route		255
Arterial	182 (71)	
Venous	14 (6)	
Orcular	2 (1)	
Multiple	57 (22)	
Adverse events		76
Cerebral or intraventricular hemorrhage	25 (33)	33
Cerebral ischemia	15 (20)	20
Hydrocephalus	1 (1)	1
Developmental delay	2 (3)	3
Thalamic syndrome	3 (4)	4
Other neurologic complication	5 (7)	7
Vessel perforation	8 (11)	11
Non-neurologic complication	4 (5)	5
Death	12 (16)	16

### Incomplete vs. complete occlusion after VOGM embolization

Data regarding the extent of occlusion after each embolization session was obtained for 77% (235/307) of participants. For participants who underwent multi-staged embolization, the extent of occlusion at their most recent embolization was used. Incomplete embolization was defined as <100% occlusion. The extent of occlusion was confirmed by digital subtraction angiography (DSA) or CT angiography scans after embolization. Incomplete and complete occlusion occurred in 48% (114/235) and 51% (121/235) of participants, respectively. Of the 90 participants with incomplete embolization where presenting symptom information was present, 72 (80%) had heart failure, 5 (6%) with hydrocephalous, 4 (4%) with seizure, 4 (4%) were from increased intracranial pressure, 2 (2%) were a prenatal diagnosis, 1 (1%) with facial pain, and the remaining patient (1%) had an undefined neurological symptom. Of the 68 participants with incomplete embolization where presenting symptom information was present, 41 (60%) had heart failure, 12 (18%) with hydrocephalous, 4 (6%) were from increased intracranial pressure, 4 (6%) from intracranial hemorrhage, 2 (3%) were a prenatal diagnosis, 1 (1%) with seizure, 1 (1%) with facial pain, 1 (1%) was an incidental finding, and the remaining 2 (3%) participants presented with undefined neurological symptoms. Information on single or multistage embolization was obtained from 99% (305/307) of participants, of which 50% had multistage embolization. There were no differences in the probability of incomplete embolization between age groups.

### VOGM embolization adverse events

Data regarding intra/post-operative adverse events was present for 25% (76/307) of participants. Adverse events included in our analysis were cerebral or intraventricular hemorrhage, cerebral ischemia, hydrocephalus, developmental delay, vessel perforation, non-neurologic complication, thalamic syndrome, other neurologic complication, and death. These data are listed in Table 1.

### Factors associated with poor clinical outcome following VOGM embolization

**Figure 2:**
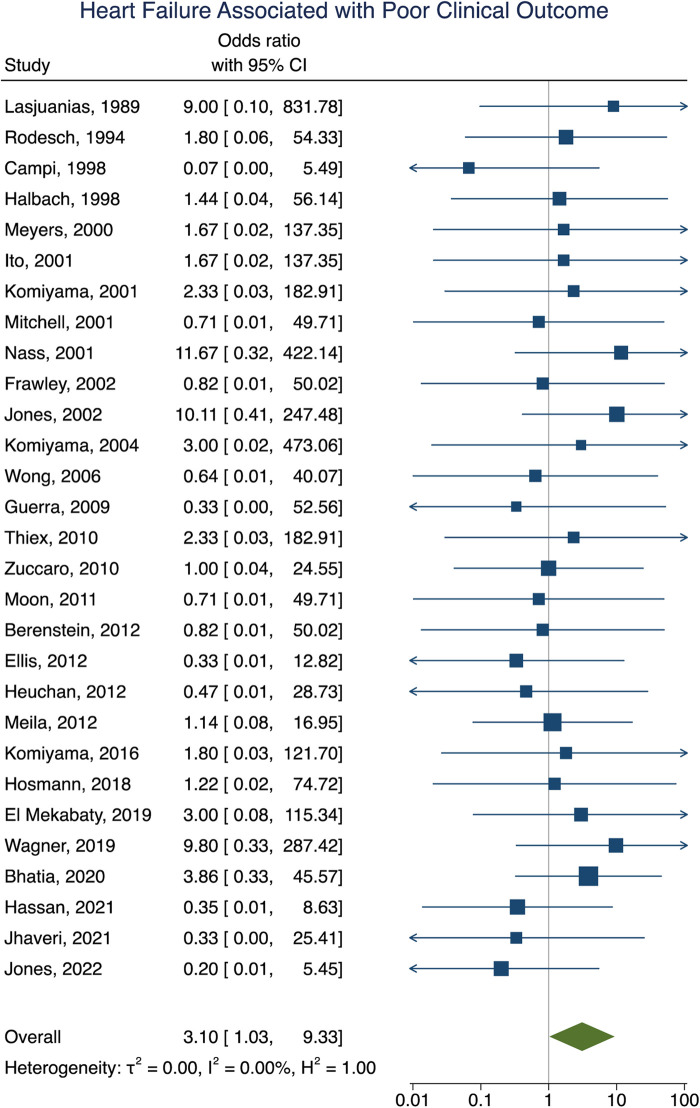
Forest plot showing the association of heart failure as a presenting symptom with poor clinical outcome after VOGM endovascular embolization.

**Figure 3:**
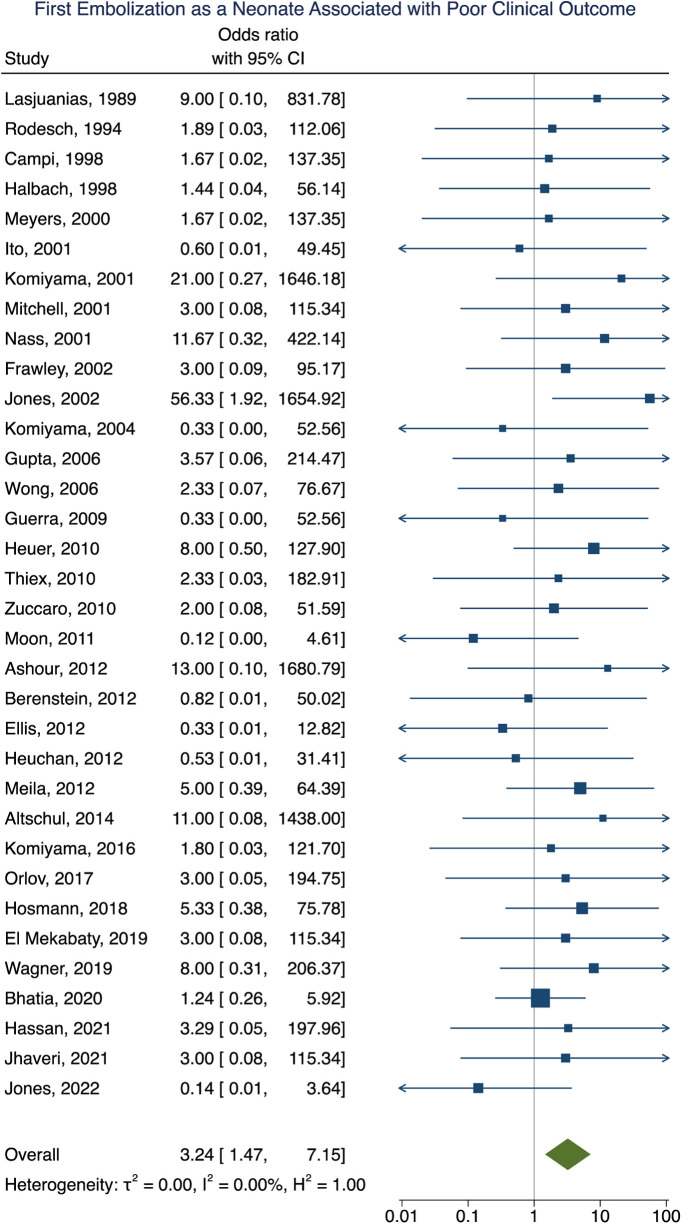
Forest plot of the association of first embolization as a neonate with poor clinical outcome after VOGM embolization.

**Figure 4:**
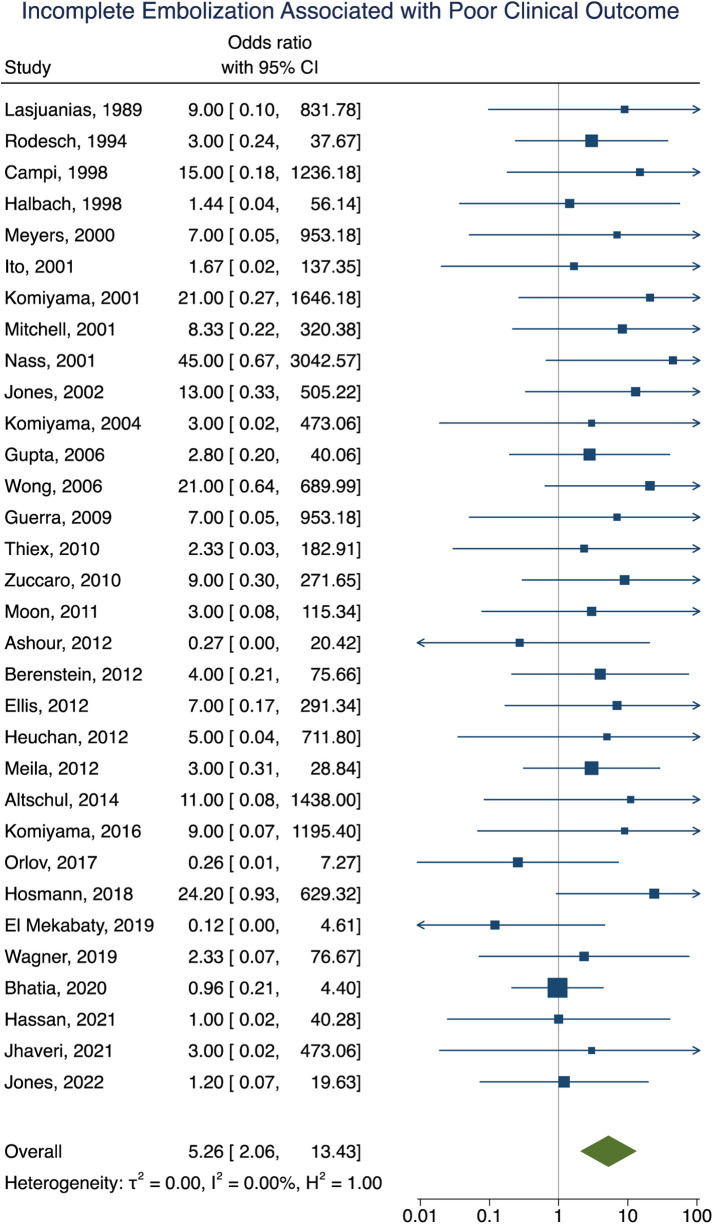
Forest plot showing the association of incomplete embolization with poor clinical outcome after VOGM embolization.

Clinical outcome data were obtained in 90% (277/307) of participants. A summary of the findings can be found in Table 2. Poor clinical outcome occurred in 32% (90/277) of participants. Participants with heart failure at presentation were significantly more likely to have a poor clinical outcome [OR = 3.10; 95% CI (1.03–9.33); *I*^2^ < 0.01; *p* = 0.002, Figure 2]. First embolization as a neonate was also found to be correlated with poor clinical outcome [OR = 3.24; 95% CI (1.47–7.15); *I*^2^ < 0.01; *p* < 0.001, Figure 3]. Further analysis showed that 85% (105/124) of neonates presented with heart failure. Participants with incomplete embolization were more likely to have a poor clinical outcome [OR = 5.26; 95% CI (2.06–13.43); *I*^2^ < 0.01; *p* < 0.001, Figure 4]. No association between sex, angioarchitecture of the lesion, single or multistage embolization, or embolization vascular access route and poor clinical outcome was found. Factors associated with mortality following VOGM embolization.

**Table 2:** Overall endovascular embolization outcomes.

Risk factor	Outcome	Overall odds ratio [95% CI]	Heterogeneity (*I*^2^)	*p*-value
Heart failure	Poor clinical outcome	3.10 [1.03–9.33]	<0.01	0.002
First embolization as a neonate	Poor clinical outcome	3.24 [1.47–7.15]	<0.01	<0.001
Incomplete embolization	Poor clinical outcome	5.26 [2.06–13.43]	<0.01	<0.001
First embolization as a neonate	Mortality	6.93 [1.99–24.08]	<0.01	<0.001
Incomplete embolization	Mortality	10.87 [1.86–63.55]	<0.01	<0.001

**Figure 5:**
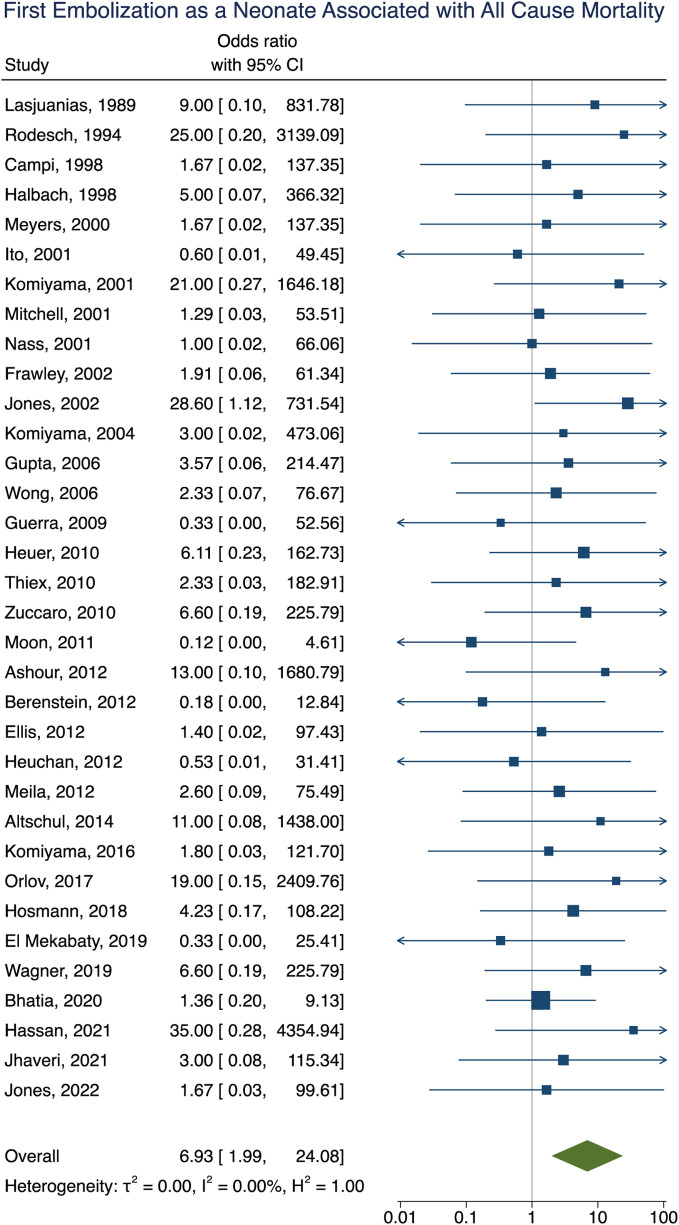
Forest plot showing the association of first embolization as a neonate with all-cause mortality after VOGM embolization.

**Figure 6:**
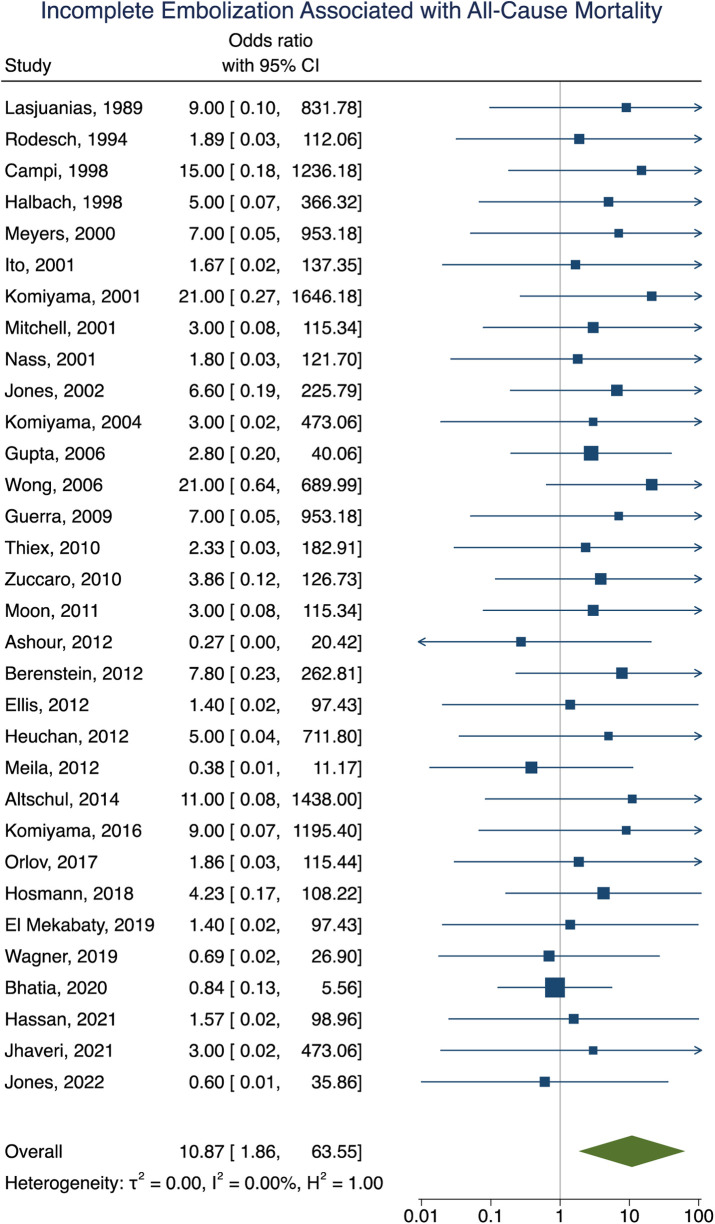
Forest plot showing the association of incomplete embolization with all-cause mortality after VOGM embolization.

Mortality data was obtained in 92% (281/307) of participants with an overall all-cause mortality rate of 16% (46/281). Of these participants, 16 died as a result of operative or perioperative intracranial hemorrhage, 9 from progressive heart failure, 9 from multiorgan failure, 5 from intraoperative vessel perforation, 4 from diffuse hypoxic-ischemic cerebral injury, 1 from meningitis, and 1 from pulmonary hemorrhage. No information for the cause of death of the remaining participant could be found. The age group of these participants included 34 neonates, 9 infants, and 3 children or adults. Participants that had their first embolization as a neonate were associated with increased rates of mortality [OR = 6.93; 95% CI (1.99–24.08); *I*^2^ < 0.01; *p* < 0.001, Figure 5]. Incomplete embolization was also significantly correlated with increased rates of mortality, with death being greater than 10-times more likely to occur in these participants [OR = 10.87; 95% CI (1.86–63.55); *I*^2^ < 0.01; *p* < 0.001, Figure 6]. Similar to our results for poor clinical outcome, no association was found between all-cause mortality and sex, angioarchitecture of the lesion, single or multistage embolization, or embolization vascular access route. Due to statistical limitations, we could not rigorously assess etiology of mortality.

## Discussion

Endovascular embolization is the current treatment modality for VOGMs (6–12, 35). However, there is limited quantitative data on risk factors associated with worse outcomes in patients undergoing VOGM embolization. In agreement with prior literature, we found that heart failure as the presenting symptom and first embolization as a neonate was associated with an increased risk of poor clinical outcome (35, 36). Also in agreement, we found first embolization as a neonate was associated with increased mortality. However, we also identified incomplete embolization as an independent factor for poor clinical outcome and mortality. Since degree of VOGM lesion occlusion is partially dependent on operative technique and treatment strategy, in addition to lesion anatomy and size among other factors, this finding may have clinical relevance. We are confident in these data since we were able to recapitulate prior associations with age and heart failure as well as very low heterogeneity between studies. However, we acknowledge there may be bias in these results given that published series represent the experience of “high” volume centers, but not necessarily all providers performing VOGM embolization.

Several factors have contributed to the lack of large-scale data on the predictors of poor outcomes following embolization of VOGMs. Given the low incidence of VOGM, it is difficult to amass a cohort large enough in any given retrospective or prospective study to assess risk factors. In addition, given the high mortality of the natural history of VOGMs and large variation of the severity of the disease at presentation, it may be difficult to control for confounding variables (e.g., angioarchitecture of lesion, severity of heart failure, technical expertise, etc.). In addition, it is common for only 1 operator to perform VOGM embolization at each institution. To date, two meta-analyses have attempted to address these issues (35, 36). Of the two, only Brinjikji et al evaluated potential factors associated with poor clinical outcomes (35, 36). However, by utilizing reported aggregate data, they were only able to compare averaged event rates taken from each study. This in turn limited their ability to control for confounding variables and identify independent risk factors associated with poor clinical outcomes.

Here we identified incomplete embolization as an independent factor associated with poor clinical outcome and mortality. It is possible that the association between incomplete VOGM embolization and poor clinical outcomes / mortality is because children could have passed away, become too medically ill, sustained irreversible cardiac failure, or suffered unsalvageable hemorrhage/stroke, precluding any additional embolizations from taking place. Alternatively, this association may be due to a conservative, staged embolization approach outlined by Lasjeunais et al. Since it was not possible to collect and analyze these data over time, this remains an open question. Yan et al. noted that those patients with complete occlusion achieved in a single stage had a higher incidence of cerebral hemorrhage and venous thrombosis in comparison to those who were treated with 2 or 3 stages (cerebral hemorrhage: 32% vs. 21%, venous thrombosis: 27% vs. 24%). It has been thought that staged embolizations may be better tolerated and with lower complication profile for patients with larger lesions (8). The rationale is that partial occlusion allows for less abrupt hemodynamic changes and enables gradual redistribution of blood flow. This idea has been particularly important in guiding treatment decisions concerning the prevention of heart failure in neonates (67). However, the data underlying these assumptions is limited as sample sizes were small and performed at a single institution. While two meta-analyses exist that investigate outcomes of embolization in VOGM, neither analyzed the association between incomplete embolization and clinical outcome and mortality (35, 36). While Yan et al. did note in patients embolized in one stage vs. 2 or 3 stages that there were higher rates of cerebral hemorrhage (32% vs. 21%) and venous thrombosis (27% vs. 24%), no statistical analysis of these results was performed. Furthermore, higher rates of complications do not directly translate to poorer clinical outcomes or mortality *per se*. In addition, Yan et al. report substantially higher heterogeneity between studies in their analysis of incomplete and complete occlusions since they were not able to utilize individual-patient data. Using the GRADE criteria (68), they also noted that the quality of the body of evidence was very low for both mortality in embolized patients and clinical outcome. In a later meta-analysis, Brinjikji et al. noted no differences in complete embolization rates in neonates compared to infants. However, further analysis of complete embolizations was limited by moderate rates of heterogeneity between studies in neonates and high rates of heterogeneity between studies in infants. As such, no analysis of the relationship between incomplete embolization and clinical outcomes or mortality was performed. Taken together, conclusions drawn from these studies regarding the association of incomplete embolization and clinical outcome are substantially limited.

The series of 216 patients by Lasjaunias et al. (9) undoubtedly had a major impact in guiding treatment for VOGM (32). In contrast to the two previous meta-analyses, we did not include the data from this cohort. In comparison, the Lasjaunias et al cohort accounted for 41% (216/532) and 37% (216/578) of the participants in the Yan et al. and Brinjikji et al. studies, respectively. This influence could be especially impactful for variables that were present in the Lasjaunias study but less commonly included in other studies. For example, in our study we were only able to obtain data of the degree of occlusion from 235 participants. As such, Yan et al. and Brinjikji et al.'s analyses of the degree of occlusion were likely heavily influenced by the Lasjaunias et al. study. Similarly, more aggressive attempts to achieve complete embolization earlier, especially in one stage, were discouraged influenced the timing and total number of embolization treatment. The impact of the Lasjaunias' work is evidenced by the creation of the Bicêtre neonatal evaluation score stratified VOGM patients by utilized information of the patient's cardiac, cerebral, respiratory, hepatic, and renal function to predict their degree of cerebral tissue impairment not evident on imaging (9). In the Brinjikji et al. meta-analysis, 23% (7/31) of the included studies, accounting for 53% (305/578) of their included participants, utilized the Bicêtre neonatal evaluation score and likely contributed to significant selection bias within their study. Similarly, of the data from 27 institutions in their analysis, 14 (52%) were found to have a high risk of bias and 7 (26%) were found a moderate risk of bias. Yan et al. also noted significant bias in their analysis, with publication bias present for 56% (10/18) of included variables [(age at treatment (neonate), clinical outcome, mortality (total mortality, mortality for the embolized patients, technical mortality, other reasons), and complications (cerebral hemorrhage, cerebral ischemia, hydrocephalus, and developmental delay)].

It is perhaps not surprising that neonates with VOGM are more likely to experience poor clinical outcomes and increased mortality. First, there are increasing technical challenges of performing VOGM embolization in neonates due to anatomical constraints. Second, neonates with VOGM are more likely to be hemodynamically unstable and medically complex necessitating emergent intervention. Owing to the nature of our study design, we were unable to control for objective measures of overall medical stability that may influence these results. These data should be incorporated into future studies of VOGM outcomes. Similarly, since neonates with VOGM have altered brain development and experience early neurologic injury, many functional neurologic outcomes may be unavoidable in such severe cases. For example, one study showed that a high degree of stenosis (>70%) of the draining sinus was significantly associated with VGAM aneurysmal enlargement and occurrence of hydrocephalus (65). Hemodynamic abnormalities in-utero could also cause hypoxic brain injury prior to endovascular embolization, leading to worse outcomes in neonates. Similarly, Jhaveri et al. found a that combined cardiac index was higher in those with significant parenchymal volume loss on MRI (66).

### Limitations

Our study has several limitations. Meta-analyses are inherently limited by the available published data, a lack of randomization, and ecologic bias, which is exacerbated here due to the predominance of single-center study design. Furthermore, all included studies within our analysis were either case-control or case series. As such, there is a risk of selection, recall, and misclassification bias given that no prospective studies were included in our analysis. Selection bias may have also been present in situations where patients were not embolized due to believed futility of treatment and thus were not included in the study's results. As such, there was lack of information on the outcomes of patients who were not embolized. There is also an increased risk of sampling bias as participants who present as a neonate typically have more severe disease, potentially limiting the generalizability of the association between incomplete embolization and mortality. As embolization technique and operator expertise has improved since its inception in the early 1980s, inclusion of older studies could cause our results to overpredict mortality rate and adverse event risk in a modern setting. No weighted analysis by year of publication was performed. In addition, given the lengthy interval between some of the included studies and the time point of data collection, there is an increased risk of recall bias and misclassification bias.

As a retrospective analysis, we can only identify an association between the analyzed variables and clinical outcome and mortality, rather than direct causation. Given that many of the included studies had a small sample size, there is an increased risk of publication bias. There may also be confounding variables that were present but not identified because they were not measured. The quality of evidence for our results may also be limited by a lack of blinding within the included studies. However, this is inherent to any analysis of VOGM embolization outcomes, given that blinding of study personnel is likely not feasible. The association between incomplete embolization and mortality may therefore be skewed as these infants might not have been medically stable for additional embolization procedures (e.g., from complications, more severe disease that didn't allow time for multistage embolizations or more aggressive embolization approaches).

### Risk of bias assessment

Included studies are at a high risk of bias since all but 1 study was single center, the largest series contained 33 patients, and the vast majority were case series study design (69). In addition, these papers likely represent the experience of high-volume centers, but not necessarily all facilities in which VOGM embolization is performed. Thus, publication bias (i.e., reporting of predominantly “good” outcomes) may be skewing our results.

## Conclusions

While this study provides the highest level of evidence for VOGM embolization to date, prospective multicenter studies in the form of a patient registry are needed to understand the optimal treatment strategies, outcomes, and natural history after VOGM embolization. We identify incomplete embolization and neonatal age as factors independently associated with poor clinical outcome and mortality. In our view, this reflects the challenge of treating patients in this age group. Thus, while outcomes may better for those who undergo intervention later in life, we not advocate for delaying treatment since the pathophysiology of VOGM in this age group may be fundamentally different. Similarly, while patients with incomplete embolization were more likely to experience poor clinical outcomes and at higher risk of death, we do not advocate for attempting complete embolization if it is not safe to do so.

## Data Availability

The raw data supporting the conclusions of this article will be made available by the authors, without undue reservation.
