# Supplementary material for: Outcomes of endovascular embolization for Vein of Galen malformations: An individual participant data meta-analysis
Source: Front Pediatr. 2022 Sep 30;10:976060. doi: 10.3389/fped.2022.976060 (PMC9561813; doi:10.3389/fped.2022.976060)
Supplement: Supplementary file 1 [file TableS1.docx]

| **Supplementary table 1.** Summary of included studies with individual-participant data | | | | | | |
| --- | --- | --- | --- | --- | --- | --- |
| **Study** | **Study type** | **Single vs multicenter** | **Number of Participants** | **Factors*** | **Outcomes** | **Mean follow up (months)** |
| **Lasjuanias, 1989** | **Case series** | **Single** | **2** | **a,c,d,f,g** | **Clinical outcome, mortality** | **-** |
| **Rodesch, 1994** | **Case series** | **Single** | **12** | **a,c,d,e,f,g** | **Clinical outcome, mortality** | **6** |
| **Borthne, 1997** | **Case series** | **Single** | **14** | **a,c,d,f,** | **N/A** | **-** |
| **Campi, 1998** | **Case series** | **Single** | **3** | **a,b,c,d,f,g** | **Clinical outcome, mortality** | **23.7** |
| **Halbach, 1998** | **Case series** | **Single** | **8** | **a,b,c,d,e,f,g** | **Clinical outcome, mortality** | **52** |
| **Meyers, 2000** | **Case series** | **Single** | **3** | **a,c,d,e,f,g** | **Clinical outcome, mortality** | **66.3** |
| **Ito, 2001** | **Case series** | **Single** | **3** | **a,b,c,d,e,f,g** | **Clinical outcome, mortality** | **19.7** |
| **Komiyama, 2001** | **Case series** | **Single** | **4** | **a,b,c,d,e,f,g** | **Clinical outcome, mortality** | **1.5** |
| **Mitchell, 2001** | **Case series** | **Single** | **5** | **a,b,c,d,e,f,g** | **Clinical outcome, mortality** | **19.2** |
| **Nass, 2001** | **Case series** | **Single** | **6** | **a,b,c,f,g** | **Clinical outcome, mortality** | **46** |
| **Frawley, 2002** | **Case series** | **Single** | **9** | **a,c,d,e,f,** | **Clinical outcome, mortality** | **22.7** |
| **Jones, 2002** | **Case series** | **Single** | **13** | **a,c,d,e,f,g** | **Clinical outcome, mortality** | **31.7** |
| **Komiyama, 2004** | **Case series** | **Single** | **1** | **a,b,c,d,e,f,g** | **Clinical outcome, mortality** | **1** |
| **Gupta, 2006** | **Case series** | **Single** | **15** | **a,d,e,f,g** | **Clinical outcome, mortality** | **75.8** |
| **Wong, 2006** | **Case series** | **Single** | **8** | **a,c,d,e,f,g** | **Clinical outcome, mortality** | **37.6** |
| **Guerra, 2009** | **Case series** | **Single** | **3** | **a,b,c,d,f,g** | **Clinical outcome, mortality** | **-** |
| **Hassan, 2010** | **Case series** | **Single** | **8** | **c,d,e,** | **N/A** | **-** |
| **Heuer, 2010** | **Case series** | **Single** | **11** | **a,b,d,e,f,** | **Clinical outcome, mortality** | **40.5** |
| **Thiex, 2010** | **Case series** | **Single** | **4** | **a,b,c,d,e,f,g** | **Clinical outcome, mortality** | **8.9** |
| **Zuccaro, 2010** | **Case series** | **Single** | **8** | **a,b,c,d,e,f,g** | **Clinical outcome, mortality** | **-** |
| **Moon, 2011** | **Case series** | **Single** | **5** | **a,b,c,d,e,f,g** | **Clinical outcome, mortality** | **26.4** |
| **Ashour, 2012** | **Case series** | **Two centers** | **6** | **a,b,e,f,g** | **Clinical outcome, mortality** | **6** |
| **Berenstein, 2012** | **Case series** | **Single** | **9** | **a,b,c,d,e,f,g** | **Clinical outcome, mortality** | **-** |
| **Ellis, 2012** | **Case series** | **Single** | **5** | **a,b,c,d,e,f,g** | **Clinical outcome, mortality** | **53.5** |
| **Heuchan, 2012** | **Case control** | **Single** | **12** | **a,c,f,g** | **Clinical outcome, mortality** | **-** |
| **Meila, 2012** | **Case series** | **Single** | **14** | **a,b,c,d,e,f,g** | **Clinical outcome, mortality** | **53** |
| **Altschul, 2014** | **Case series** | **Single** | **5** | **a,b,f,g** | **Clinical outcome, mortality** | **-** |
| **Komiyama, 2016** | **Case series** | **Single** | **6** | **a,b,c,d,e,f,g** | **Clinical outcome, mortality** | **6.2** |
| **Orlov, 2017** | **Case series** | **Single** | **9** | **a,b,d,e,f,g** | **Clinical outcome, mortality** | **9.7** |
| **Hosmann, 2018** | **Case series** | **Single** | **17** | **a, b, c, d, e, f, g** | **Clinical outcome, mortality** | **149.4** |
| **El Mekabaty, 2019** | **Case series** | **Single** | **5** | **a, b, c, d, e, f, g** | **Clinical outcome, mortality** |  |
| **Wagner, 2019** | **Case series** | **Single** | **8** | **a, b, c, d, e, f, g** | **Clinical outcome, mortality** | **14.2** |
| **Bhatia, 2020** | **Case series** | **Single** | **33** | **a, b, c, d, e, f, g** | **Clinical outcome, mortality** | **60.6** |
| **Hassan, 2021** | **Case series** | **Single** | **17** | **a, c, d, e, f, g** | **Clinical outcome, mortality** | **21** |
| **Jhaveri, 2021** | **Case control** | **Single** | **5** | **a, c, e, f, g** | **Clinical outcome, mortality** | **20.6** |
| **Jones, 2022** | **Case series** | **Single** | **11** | **a, b, c, d, e, f, g** | **Clinical outcome, mortality** | **38.5** |
| Factors reported* a) age, b) sex, c) presenting symptom, d) angioarchitecture of lesion, e) embolization technique, f) number of embolizations, g) degree of occlusion. | | | | | | |
